# An Analysis of the *Malassezia* Species Distribution in the Skin of Patients with Pityriasis Versicolor in Chengdu, China

**DOI:** 10.1155/2014/182596

**Published:** 2014-08-10

**Authors:** Zhen Xie, Yuping Ran, Hao Zhang, Min Zhang, Huiying Wan, Conghui Li

**Affiliations:** ^1^Department of Dermatology, West China Hospital, Sichuan University, Chengdu 610041, China; ^2^Department of Dermatology, Sichuan Academy of Medical Science & Sichuan Provincial People's Hospital, Chengdu 610072, China; ^3^Department of Gastroenterology, The Sixth Affiliated Hospital, Sun Yat-sen University, Guangzhou 510000, China

## Abstract

Pityriasis versicolor (PV) is a common clinical problem associated with *Malassezia* species (*Malassezia* spp.). Controversies remain regarding the specific species involved in the development of PV. This study analyzed the difference in *Malassezia* spp. distribution in lesional and nonlesional skin in Chinese PV patients. A paired design was applied. Lesional and nonlesional scales from 24 cases were collected; real-time fluorescence quantitative PCR was used to detect 10 different *Malassezia* spp. In lesional skin, the highest detection rates were for *M. globosa* (95.8%), *M. restricta* (91.7%), and *M. sympodialis* (50.0%). In nonlesional skin, the highest detection rates were for *M. globosa* (87.5%), *M. restricta* (79.2%), and *M. dermatis* (33.3%). A significant difference in the detection rate was only found for *M. sympodialis* (50.8% versus 20.8%, *P* = 0.04). Compared with nonlesional skin, the amount of *M. globosa*, *M. restricta*, and *M. sympodialis* in lesional skin was significantly higher (3.8 ± 1.3,  2.5 ± 1.1, and 3.2 ± 1.6 times higher, resp.). The results of this study do not indicate that *M. globosa* and *M. restricta* are directly correlated with PV development; however, *M. sympodialis* is more likely related to PV development in Chinese individuals.

## 1. Introduction

The lipid-dependent yeast* Malassezia* species (*Malassezia* sp.) is widely found on the skin surface of humans and other animals [1, 2]. This fungus can cause pityriasis versicolor,* Malassezia* folliculitis, and seborrheic dermatitis. Additionally, its infection can worsen the condition of immunocompromised infants and young children [3–6].

It was recognized in 1846 that the pathogen of pityriasis versicolor (PV) was a fungus [7], but it was first named as the genus* Malassezia* by Baillon until 1889. By 2011, 14 different* Malassezia* spp. had been differentiated by morphology, biochemistry, rDNA sequencing, restriction fragment length polymorphism (RFLP), and other methods. These species include* M. furfur*,* M. sympodialis*,* M. obtusa*,* M. globosa*,* M. restricta*,* M. slooffiae*,* M. pachydermatis*,* M. dermatis* [8],* M. japonica *[9],* M. yamatoensis* [10],* M. nana* [11],* M. caprae*,* M. equina* [12], and* M. cuniculi *[13]. There have been reports of* M. pachydermatis *in humans [4, 14], whereas* M. nana*,* M. caprae*,* M. equina*, and* M. cuniculi* are pro-animal species that have never been detected in humans. The other species exist in human skin with varying distributions across species depending on the location and the local microenvironmental variation.

To date, controversies remain over the specific species involved in the development of PV, and the results from different countries are not consistent. In general, it is believed that* M. globosa* is the main species correlating with PV development, and this idea is mainly based on the analyses of the predominant species of lesional skin in the Japanese population [15, 16]. In other districts and regions, such as Greece and Spain, similar reports have also been found [17, 18]. In some studies that analyzed species differences between lesional and nonlesional skin, the detection rate of* M. globosa* in lesional skin was higher compared with nonlesional skin [17, 19]. However, there was also* Malassezia* spp. distribution in nonlesional skin of healthy individuals and PV patients, with extremely high detection rates in some studies; for example, the detection rate of* Malassezia* in healthy Japanese individuals was as high as 86.7% [20]. Moreover, Miranda et al. showed that* M. furfur* was the predominant species in the lesional skin of PV patients in Brazil [21], whereas Gupta et al. showed in a Canadian population and Ramadán et al. showed in Argentineans that* M. sympodialis* was more common in PV patients [22, 23]. In Gupta's study, the detection rate of* M. globosa* in lesional skin was only 6.3% [22]. The differences in these studies might be related to the regional differences of* Malassezia* distribution [24–26]. Although PV is common in China [27], there have not been reports on* Malassezia* spp. distribution in the Chinese PV population to date.

Based on these findings, we conducted the current study to analyze the constituents of* Malassezia* spp. in lesional skin and nonlesional skin in Chinese PV patients using real-time fluorescence quantitative polymerase chain reaction (RT-PCR). The results of this study may shed light on the pathogenesis of PV.

## 2. Materials and Methods

### 2.1. Study Subjects

Twenty-four patients with PV from an outpatient clinic consecutively entered this study from April 2010 to July 2010. PV was diagnosed by its clinical appearance and the staining of scales with 20% KOH and Parker blue ink in microscopic examination. The inclusion and exclusion criteria included the following: (1) typical clinical manifestations: pigmentation or hypopigmentation spots on the body trunk or the forearm; (2) lesional scales that showed thick and short hyphae and round or oval spores under the microscope with methylene blue staining; (3) no antifungal agents administered orally or externally within one month before the visit; (4) no* Malassezia*-related diseases, such as* Malassezia* folliculitis, atopic dermatitis, or seborrheic dermatitis. The clinical diagnoses of the patients were made by the same dermatologist.

All enrolled patients signed an informed consent form. All procedures were in accordance with the Declaration of Helsinki and were approved by the Ethics Committee of the Sichuan Academy of Medical Science & the Sichuan Provincial People's Hospital, China.

### 2.2. Scale Sample Collection and Genome DNA Extraction

For each patient, 1 lesional scale sample and 1 nonlesional scale sample were collected. The samples were collected using a previously published method [28].* Malassezia *samples were collected by applying Tegaderm transparent dressings (6 × 7 cm; 3 M Health Care, Neuss, Germany) on the target skin of patients with PV. The collected transparent dressings were placed in a 1% Triton X-100 solution, and the suspensions were transferred into sterile Eppendorf tubes. The supernatants were removed by centrifugation at 4,000 ×g. The sample collection was conducted by the same dermatologist, and the lesional scale samples were obtained from thoracic lesions to reduce the impact of the location on the distributions of the species.* Malassezia* spp. are lipid-dependent yeasts that mainly colonize skin that easily develops seborrhea, such as the chest, back, and face. We collected samples of thoracic lesions and of distant nonlesional skin from the same PV patient and performed comparisons to reduce the influence of the skin microenvironment of different individuals on* Malassezia* colonization. Because* Malassezia* hyphae are found on skin near PV skin lesions [19], skin distant from the lesions was selected as the area of nonlesional skin sampling in this study. In these areas, hyphae were not detected. A total of 10* Malassezia* reference strains that are maintained in our laboratory were utilized as positive controls [28]. Sampling and DNA extraction followed methods previously described in the literature [28] and the OMEGA D3370-01 kit instructions.

### 2.3. Verification of DNA Extraction through PCR with Universal Fungal Primers

The primers were universal fungal primers: ITS1 (5′-TCCGTAGGTGAACCTGCG-3′) and ITS4 (5′-TCCTCCGCTTATTGATATGC-3′). The constituents of the reaction system and the total volumes are summarized in Table 1. PCR, electrophoresis, and other laboratory tests were all performed using a blind method.

The reaction conditions consisted of predenaturation for 5 min at 94°C, 35 cycles of 94°C for 45 s, 58°C for 45 s, and 72°C for 45 s, and a final step at 72°C for 7 min. The PCR products were processed by 1.5% agarose electrophoresis for 35 min, and the results were observed under UV light and photographed. A negative control was tested during each amplification. The amplified products were considered valid only when there was no DNA band generated after the amplification of the negative control.

### 2.4. Extracted DNA Concentration as Determined by Spectrophotometry

Extracted DNA concentrations from the lesional and nonlesional skin were determined by spectrophotometry to determine whether there were different concentrations in the lesion and nonlesion groups and to ensure that the initial concentrations of the templates for real-time fluorescence quantitative PCR were the same. If the initial concentrations of the two groups were not the same, corrections were made by dilution. The TE solution that contained dissolved DNA was set as the blank control. One microliter of sample was drawn by a micropipette and dropped onto the test platform, and the absorbance and concentration of the sample were determined (*μ*g/*μ*L).

### 2.5. RT-PCR

The design of the primers and the probes was based predominately on the ITS sequences of 10* Malassezia* species released by GenBank. Primer 5.0 software was used to design the* Malassezia*-specific primers, the species-specific primers, and their corresponding TaqMan fluorescent probes to amplify the* Malassezia* species and the 10 different* Malassezia* target DNAs. The specific sequences are shown in Table 2. The 48 DNA samples underwent fluorescence quantitative PCR and reacted with the* Malassezia* genus-specific primer/probe and the 10 sets of* Malassezia* primers/probes. The qualitative results (positive bands) and quantitative results (Ct values) were recorded.

The reaction was carried out in a 0.2 mL EP tube in 30 *μ*L of reaction volume. The components are summarized in Table 3. The PCR conditions consisted of a predenaturation step at 94°C for 2 min, 45 cycles of 94°C for 20 s, 50°C for 20 s, and 60°C for 30 s, and a final extension step at 72°C for 7 min.

The amplification cycle number when the fluorescence intensity reached a certain threshold (Ct value) in each sample tube was determined according to the kinetic curve. Water was used as the template and the negative control during each amplification. The amplified products were considered valid only when there was no DNA band generated after the amplification of the negative control.

### 2.6. Specific Verification Using* Malassezia* Genus-Specific Primers and Species-Specific Primers

*Malassezia* reference strains were used as the positive controls, and fluorescence quantitative PCR products were collected. The products were sent to Shanghai Biological Engineering Co., Ltd., with their corresponding primers to be sequenced for verification. The results were compared with the sequences from GenBank (http://blast.st-va.ncbi.nlm.nih.gov/Blast.cgi), and the specificity of the primers was determined based on this comparison.

### 2.7. Statistical Analysis

SPSS13.0 (Chicago, IL, USA) was utilized in the statistical analyses. Data with normal distributions are presented as the $\overset{-}{X}\pm S$ (mean ± standard deviation), and the enumeration data are expressed as percentages. A  *χ* ^2^ test was used in the comparison of species detection rates of lesional skin and nonlesional skin, and a *t*-test was used in the quantitative comparison of species in lesional skin and nonlesional skin; a two-tailed *P* value < 0.05 was regarded as a statistically significant difference.

## 3. Results

### 3.1. ITS Amplification Results

**Figure 1:**
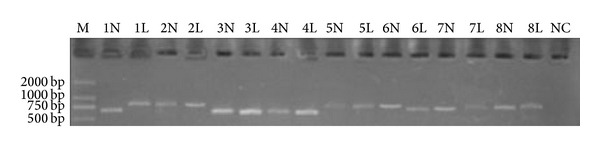
Electropherogram of the lesional and nonlesional skin DNA samples from patients 1–8 after ITS1/ITS4 amplification. The lanes of the lesional scales are coded as the patient number + L (lesion), and the lanes of the nonlesional skin samples are coded as the patient number + N (normal). M signifies the marker, and NC signifies the negative control. Positive bands appeared at the 600–800 bp position in each sample lane. No band appeared in the NC.

The ITS1/ITS4 amplification of the lesional and nonlesional skin scale DNA samples from patients 1–24 is shown in Figure 1. All 48 samples from 24 patients in this study demonstrated positive bands at 600–800 bp after ITS1/ITS4 universal fungal primer amplification.

### 3.2. *Malassezia* Species Distribution

#### 3.2.1. *Malassezia* Species in Lesional and Nonlesional Skin

First, we determined whether more* Malassezia* spp. were detected in lesions than in nonlesional skin. The detection rates of* Malassezia* spp. in lesional and nonlesional skin from the same sampling area are shown in Table 4; the detection rates were similar (3.2 ± 0.7 versus 3.4 ± 0.7, *P* = 0.208), indicating that the number of* Malassezia* species detected did not correlate with the development of PV.

**Figure 2:**
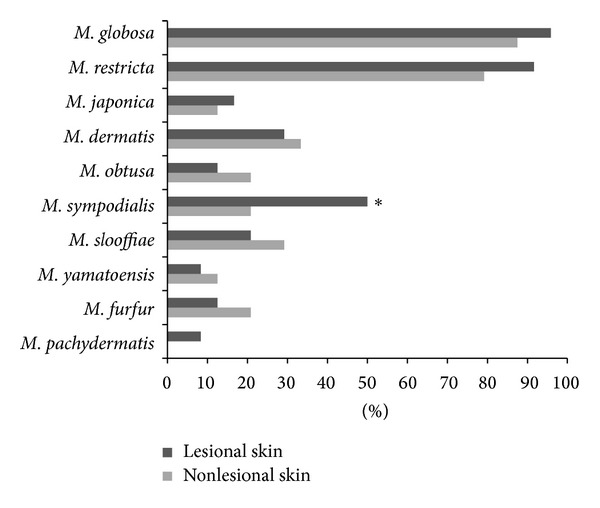
*Malassezia* species detection rates in the lesional and nonlesional skin of the 24 patients. ∗*P* < 0.05, nonlesional skin versus lesional skin.

The detection rate of each* Malassezia* species is shown in Figure 2. Ten* Malassezia* species were detected in the lesional skin, and 9* Malassezia* species were detected in the nonlesional skin of the 24 patients with PV.* M. globosa* possessed the highest detection rates in both the lesional and nonlesional skin, which were 95.8% and 87.5%, respectively. Qualitatively,* M. globosa* had the highest detection rate, followed by* M. restricta*, with rates of 91.7% and 79.17%, respectively. In the lesional skin, 10 species of* Malassezia *were detected, while* M. pachydermatis *was not detected in the nonlesional skin. In the lesional skin, the first three species of the highest detection rates were* M. globosa* (95.8%),* M. restricta* (91.7%), and* M. sympodialis* (50.0%), respectively. In the nonlesional skin, the first three species of the highest detection rates were* M. globosa* (87.5%),* M. restricta* (79.2%), and* M. dermatis* (33.3%), respectively. Among the 9 species detected in both the lesional and the nonlesional skin, a significant difference in the detection rate was only found in* M. sympodialis* (50.8% versus 20.8%, *P* = 0.04) (Figure 2). The compositions of the mixed* Malassezia* spp. detected in the same area were mainly* M. globosa* with* M. restricta* and* M. globosa* with* M. sympodialis*. Compared with nonlesional skin, the rates of simultaneously detecting* M. globosa* with* M. restricta* (91.7% versus 66.7%, *P* = 0.033) and* M. globosa* with* M. sympodialis* (4.2% versus 50%, *P* < 0.001) were higher in lesional skin, whereas there was no difference in the rates of simultaneously detecting* M. globosa* with the other 6 species in lesional versus nonlesional skin.

#### 3.2.2. Quantitative Analysis of* Malassezia* Species in Lesional and Nonlesional Skin

**Figure 3:**
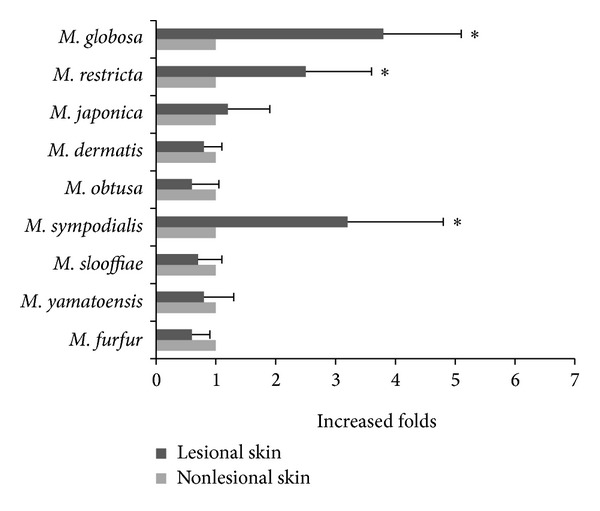
The relative quantity of each* Malassezia* species in the lesions compared with the nonlesional skin of the 24 patients. The *y*-axis displays the increased prevalence of the species in the lesions compared with the nonlesional skin. ∗*P* < 0.05, nonlesional skin versus lesional skin.

Lesional and nonlesional skin scale samples from the 24 patients with PV were amplified by fluorescence quantitative PCR using* Malassezia* genus-specific primers and 10 species-specific primers. The quantity of* M. globosa*,* M. restricta*, and* M. sympodialis* in the lesions was significantly higher compared with nonlesional skin (Figure 3); there was no significant difference in the quantity of the other* Malassezia* spp. between the groups.

## 4. Discussion

PV is a relatively common clinical condition, which is associated with the* Malassezia* spp. However, the specific species of the fungus involved in the pathogenesis of PV remains controversial.* M. globosa* and* M. restricta* are the predominant* Malassezia* spp. in PV patients; however, the* Malassezia* spp. distribution in the Chinese population remains unclear. In this study, we analyzed the distribution of the* Malassezia* spp. in both lesional and nonlesional regions of the Chinese PV patients.

*Malassezia* sp. is a global resident flora on the skin surfaces of humans and animals, which can cause PV,* Malassezia* folliculitis, and seborrheic dermatitis, and its complicated infection can worsen the condition of immunocompromised infants and young children [4–6]. Previously, a culture method was primarily adopted to investigate the distribution of* Malassezia* spp.; unfortunately, this method is easily affected by the variation of microbial growth rates and culture media. In recent years, transparent dressings have been used to make scales stick to them, and DNA extraction from the scales for real-time fluorescence quantitative PCR has become a common technique to investigate* Malassezia* rapidly and efficiently [28, 29]. Using these methods, 14* Malassezia* spp. have been identified to date, with 9 species predominately existing on human skin. Among the other 5 species that exist on animal skins,* M. pachydermatis *is predominately found in animals but can be transmitted to humans by contact with pets. Although the* Malassezia* spp. have different sensitivities to antifungal agents [30, 31], it is impossible to select highly sensitive medications to treat PV because of the incomplete understanding of the pathogenic species. The recurrence of PV remains a problem in clinical practice [24]; therefore, the identification of pathogenic species is helpful for selecting sensitive drugs in the clinic.

This is the first study to analyze the* Malassezia* spp. constituents in Chengdu PV patients. In this study, we used culture-independent real-time fluorescence quantitative PCR to successfully isolate the 10* Malassezia* spp. that exist in humans. Our results showed that, among the 10 detected species,* M. sympodialis*, with the third highest detection rate, was the only species that had a significantly higher detection rate and a larger quantity in the lesions compared with nonlesional skin. This is consistent with the findings by Crespo Erchiga et al. in Canada regarding PV patients [17].* M. globosa* and* M. restricta* are the two species with the highest detection rates [17, 32, 33], although there was no difference in their detection rates in lesions versus nonlesional skin (Figure 2): the quantity of these species was higher in lesions than in nonlesional skin (Figure 3). Multiple* Malassezia* spp. were detected in the same sampling area (Table 4), but due to lack of previously published results on the number of detected* Malassezia* spp. we cannot determine whether this is a characteristic distribution of* Malassezia* spp. in Chinese PV patients; this finding may relate to the use of highly sensitive PCR in this study. Therefore, it is possible that multiple coexistent* Malassezia* spp. synergize to induce PV. We analyzed the differences in the coexistence of two species in lesions versus nonlesional skin and determined that the coexistence of* M. globosa* with* M. restricta* and of* M. globosa* with* M. sympodialis* was more common in lesions than in nonlesional skin, which supported the above hypothesis.

There are some limitations to this study. We did not include healthy people as blank controls for the consideration of the possible influence of individual idiosyncrasy on the results obtained in this study. The subjects in this study only represent the population in certain Chinese cities; because regional factors may influence the distribution of* Malassezia* spp. [24–26], our conclusions cannot be extrapolated to other regions. The relatively small sample size is another limitation of this study; because there are numerous combinations of more than two coexisting species, the number of samples in each combination is insufficient for further statistical analysis. Therefore, a larger sample size is necessary for future studies. However, as shown in Figure 2, the detection rate of other species was low, with the exception of* M. globosa*,* M. restricta*, and* M. sympodialis*. Therefore, we presume that the less common* Malassezia* spp. are not significant for the development of PV.

In summary, our findings suggest that* M. sympodialis* might be involved in the development of PV in China, and its mechanism of action deserves further study. Furthermore, mixed infections, such as* M. globosa* and* M. restricta* or* M. sympodialis*, might be involved in the pathogenesis of PV.

## Figures and Tables

**Table 1:** Universal fungal primer PCR reaction system.

Components	Sample amount
PCR reaction mixture	12.5 *μ*L (including 2.5 U *Pfu Taq* enzyme, 500 *μ*M dNTP, 20 mM Tris-Cl (pH 8.3), 100 mM KCl, and 3 mM MgCl_2_)
ITS1^a^	1 *μ*L
ITS4^b^	1 *μ*L
Template DNA	2 *μ*L
Double distilled water	8.5 *μ*L

^a^Internal transcribed spacer 1; ^b^internal transcribed spacer 4.

**Table 2:** Primers and probes used in RT-PCR.

Species	Sense primer (5′ → 3′)	Antisense primer (5′ → 3′)	TaqMan probe
*Malassezia *	GTAGACTCCATCTAAAGCTAAAT	CTTTTAACTCTCTTTCCAAAGT	CCCTCACGGTACTTGTTCGCT
*M. furfur *	GTGAATTGCAGAATTCCGTGAAT	GAGCCTGTTTCTTGCGAAACA	CTTTGAACGCACCTTGCGCTC
*M. globosa *	GTGAATTGCAGAATTCCGTGAAT	GAGCTTTTTCTAGAGAAGAAAAG	CTTTGAACGCACCTTGCGCTC
*M. obtusa *	GTGAATTGCAGAATTCCGTGAAT	GCGAGCCTGTTTAGCAAGAAA	CTTTGAACGCACCTTGCGCTC
*M. pachydermatis *	GTGAATTGCAGAATTCCGTGAAT	GAGCCTGTAGTTTCCCACAG	CTTTGAACGCACCTTGCGCTC
*M. restricta *	GTGAATTGCAGAATTCCGTGAAT	GCGAGCCTGTGCTAGGTA	CTTTGAACGCACCTTGCGCTC
*M. slooffiae *	GTGAATTGCAGAATTCCGTGAAT	CTTTTCGAGCGAGCCTACCAA	CTTTGAACGCACCTTGCGCTC
*M. sympodialis *	GTGAATTGCAGAATTCCGTGAAT	TACAATCCCCAGGCAGCAA	CTTTGAACGCACCTTGCGCTC
*M. yamatoensis *	GTGAATTGCAGAATTCCGTGAAT	GCCAGCCTCGCAAGGCAT	CTTTGAACGCACCTTGCGCTC
*M. japonica *	GTGAATTGCAGAATTCCGTGAAT	TGTACGAGACACTGGCAGGCA	CTTTGAACGCACCTTGCGCTC
*M. dermati*s	GTGAATTGCAGAATTCCGTGAAT	GTTTCCCAGGCAGCGGCA	CTTTGAACGCACCTTGCGCTC

**Table 3:** Reaction system for real-time fluorescence quantitative PCR.

Components	Sample amount
10 × buffer (without Mg^2+^)	3 *μ*L
MgCl_2_ (25 mM)	3 *μ*L
dNTP (25 mM)	0.36 *μ*L
Sense prime (10 *μ*M)	1 *μ*L
Antisense primer (10 *μ*M)	1 *μ*L
TaqMan probe (10 *μ*M)	0.6 *μ*L
*Taq* enzyme (5 U/*μ*L)	0.3 *μ*L
Double distilled water	18.74 *μ*L
Template DNA	2 *μ*L

**Table 4:** Detection rates of multiple *Malassezia* spp. in the same sampling area (%).

	2 *Malassezia* spp. detected	3 *Malassezia* spp. detected	4 *Malassezia* spp. detected
Nonlesional skin	8.3	41.7	50.0
Lesional skin	16.7	50.0	33.3
